# Intelligent Point-of-Care Biosensing Platform Based on Luminescent Nanoparticles and Microfluidic Biochip with Machine Vision Algorithm Analysis

**DOI:** 10.1007/s40820-025-01745-w

**Published:** 2025-04-14

**Authors:** Yuan Liu, Xinyue Lao, Man-Chung Wong, Menglin Song, Yifei Zhao, Yingjin Ma, Qianqian Bai, Jianhua Hao

**Affiliations:** 1 Department of Applied Physics, The Hong Kong Polytechnic University, Kowloon, 999077 Hong Kong People’s Republic of China; 2 Research Centre for Nanoscience and Nanotechnology, The Hong Kong Polytechnic University, Kowloon, 999077 Hong Kong People’s Republic of China

**Keywords:** Point-of-care, Luminescent nanoparticles, Biochip, Machine vision, Biosensing

## Abstract

Realizing the point-of-care tumor markers biodetection with good convenience and high sensitivity possesses great significance for prompting cancer monitoring and screening in biomedical study field. Herein, the quantum dots luminescence and microfluidic biochip with machine vision algorithm-based intelligent biosensing platform have been designed and manufactured for point-of-care tumor markers diagnostics. The employed quantum dots with excellent photoluminescent performance are modified with specific antibody as the optical labeling agents for the designed sandwich structure immunoassay. The corresponding biosensing investigations of the designed biodetection platform illustrate several advantages involving high sensitivity (~ 0.021 ng mL^−1^), outstanding accessibility, and great integrability. Moreover, related test results of human-sourced artificial saliva samples demonstrate better detection capabilities compared with commercially utilized rapid test strips. Combining these infusive abilities, our elaborate biosensing platform is expected to exhibit potential applications for the future point-of-care tumor markers diagnostic area.

**Supplementary Information:**

The online version contains supplementary material available at 10.1007/s40820-025-01745-w.

## Highlights

- A novel intelligent biosensing platform consisting of quantum dots luminescence and biochip with machine vision algorithm is proposed for point-of-care carcinoembryonic antigen (CEA) protein diagnostics.
- The designed diagnostic platform possesses outstanding detection limitation of approximately 0.021 ng mL^−1^ of CEA concentration compared with some commercial biodetection devices of lateral flow assay strips.
- The utilization of machine vision algorithm improves the detection features of portability and integration, which expands the potential of point-of-care biosensing applications.

**Supplementary Information:**

The online version contains supplementary material available at 10.1007/s40820-025-01745-w.

## Graphical abstract

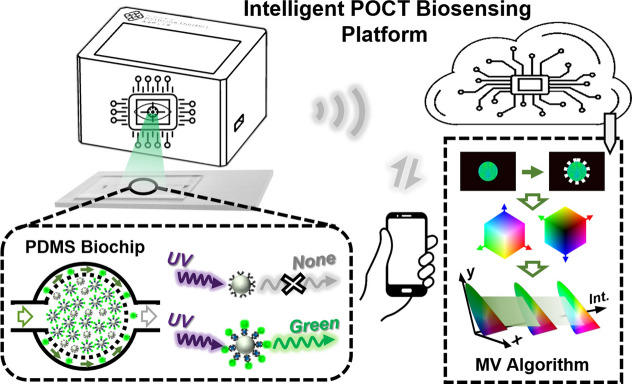

## Introduction

Globally, cancer has always been a hard-to-cure disease, which seriously affects the health and life of human beings [1–3]. In recent years, lung cancer has been regarded as a frequently diagnosed and major cause of cancer-relevant death, which resulted in approximately 1.8 million deaths during the year 2022 [4]. Several important tumor markers involving CYFRA21-1, CA125, and SCC-Ag are commonly employed for assessment of non-small-cell lung cancer, which illustrates around 85% of lung cancer detection [5]. Moreover, pancreatic cancer is usually recognized as the king of carcinoma, representing highly lethal disease with great difficulty for therapy, in which the CA 19–9 tumor marker is validated and widely utilized for pancreatic cancer diagnosis [6, 7]. Therefore, realizing a sensitive and precise detection of related tumor markers possesses significant importance for early cancer screening and diagnosis.

Radiology techniques, like computed tomography and magnetic resonance imaging scans, are often used for in vivo imaging diagnostics of cancer [8]. These methods can assist doctors in acquiring better views of diseased parts for appropriate treatment selection, but also possess some drawbacks to restrict their application, such as high detection cost, bulky instrument requirements, and professional technician operation. On the other hand, some rare earth ions-doped luminescent materials are utilized for in vivo imaging detection; however, the corresponding biotoxicity complexity of these functionalized materials still limits the application prospect [9, 10]. In that case, the relevant investigations of in vitro tumor marker diagnosis reveal great significance for convenient cancer evaluation.

Currently, tissue biopsy is relatively irreplaceable for identifying the tumor features, which is selected as a gold standard for cancer in vitro diagnostics, but this method will bring about a traumatogenic sampling and complex pathological tissue analysis, which restricts its potential applications [11]. The enzyme-linked immunosorbent assay (ELISA) is widely treated as a gold standard for protein detection, which is also employed for tumor marker diagnosis. The ELISA is more convenient for related tumor markers proteins detection; however, some existent disadvantages of time-consuming and technical operation limit the point-of-care application for tumor markers diagnosis [12]. In addition, the lateral flow assay (LFA) strip can be also utilized for point-of-care tumor marker proteins detection area [13]. Although the excellent performances of simpleness and convenience expand its application scenarios, the problems of relatively low detection sensitivity still need to be resolved. Among these tumor marker protein detections of ELISA and LFA, the blood samples are commonly used for related tests, nevertheless, the involved procedure for obtaining blood samples is invasive, which will bring about some risks of infection [14]. On the other hand, saliva samples are noninvasive and convenient for acquiring, which are well-suitable for point-of-care tumor markers diagnostics [15–17]. For instance, Kaur et al. from Indian Institute of Technology reported the related biomarkers of IL-6 and IL-8 in saliva samples for oral cancer detection [18]. According to previous research, there is around 99.5% water content in saliva samples, and the related tumor markers concentrations are much lower than the blood samples, which manifests that it is significant for high sensitivity detection of tumor markers.

Quantum dots (QDs) possess outstanding photoluminescence capabilities consisting of broad excitation wavelengths, narrow emission profile, brighter illumination property, and fairly high photoluminescence quantum yield [19–23]. Owing to these prominent luminescent abilities, the QDs have been employed in the in vitro biomarker detection field [24–26]. For instance, Prof. Chan et al. utilized the modified fluorescent QDs for multiplexed pathogens biosensing and highly sensitive genetic targets detection [27]. Moreover, the fluorescent immunoassay with sandwich structure conjugation is broadly utilized for the in vitro biomarkers diagnosis area, in which the luminescent nanoparticles are involved in the targeted biomarkers optical sensing [28–32]. The microfluidic biochip as a functionalized microdevice contains numbers of patterned channels with specific property, which enables the controlled fluids to pass through variant micro-channels and establish relevant interaction with external system under the inlet and outlet. Several advantages of microfluidic biochips are revealed involving versatility of application, programmable functionality, and biology applicability, which immensely improve the convenient and integrated performance of the designed point-of-care biosensing platform [33–36]. For example, the flexible sensing array combined with the microfluidic biosensing platform is utilized for the corresponding cancer-related biomarkers point-of-care detection [37]. In addition, the emerging machine vision technology is considered as one type of digital technology, which is employed for objective perception and visual performance simulation of human beings [38–40]. With the development of computer science, the machine vision algorithm can inform relatively strict and reliable test results under the speedy programs processing and analysis for acquired digital pictures, which is extraordinarily beneficial for the adhibition of intelligent point-of-care biosensing platform [41–44]. Besides, the involvement of robust computing can handle and eliminate the feasible outliers or errors of obtained data, which improves the accurate and quantified capabilities to a great extent for designed biosensing platform [45, 46]. Therefore, it is of great importance to combine these admirable superiorities and balance the existing advantages and disadvantages for realizing the integrated, convenient, and intelligent point-of-care biosensing platform for in vitro tumor markers diagnostics.

In this work, a novel intelligent biosensing platform involving QDs luminescence and biochip with machine vision algorithm is proposed for point-of-care tumor markers in vitro diagnostics. The bright QDs were successfully modified for the conjugation of related antibodies as the labeling agents of sandwich structure immunoassay. The utilized microfluidic biochip was well-designed and manufactured with splendid performances, which promoted the sensitivity and accessibility for point-of-care detection. The common-used tumor marker of carcinoembryonic antigen (CEA) protein serves as the diagnostic object for evaluating the detection performance of designed biosensing platform. The corresponding test results illustrate that the designed diagnostic platform possesses outstanding detection sensitivity with approximately 0.021 ng mL^−1^of tumor marker concentration compared with some commercial biodetection device of LFA strips. And the employing of machine vision algorithm improves the detection features of portability and integration, which expands the potentials of point-of-care biosensing applications. Moreover, relevant human-sourced artificial saliva samples were utilized for evaluating the practical application capabilities. As a result, these various remarkable abilities imply that the proposed and well-designed intelligent biosensing platform possesses broad prospects for point-of-care tumor markers diagnosis applications.

## Experimental Section

To investigate the intelligent biosensing platform for point-of-care tumor biomarker in vitro diagnostics, the corresponding modified quantum dots and PS microspheres were prepared, followed by the relevant morphology study, modification property, and photoluminescence performance investigation. Moreover, the related information including chemicals, materials, involved characterizations, conjugation of antibodies with quantum dots and PS microspheres, and fabrication processes of utilized microfluidic biochips are also described in the Supplementary Material.

## Results and Discussion

### Mechanism of Intelligent Biosensing Platform

**Fig. 1:**
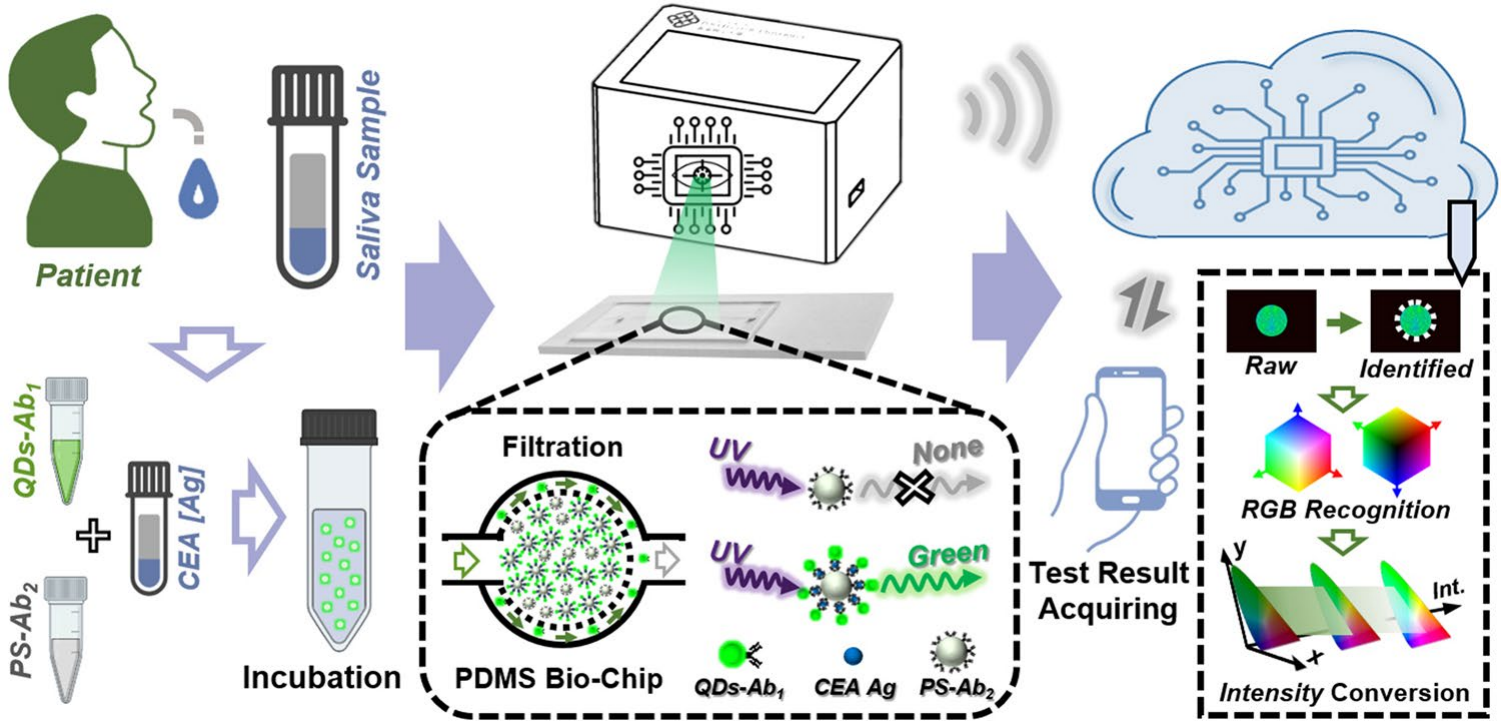
Schematic diagram of tumor marker detection platform, which consists of saliva sample collection, sandwich-based immunoassay reagents incubation, microfluidic biochip filtration for unconjugated QDs and luminescent images acquired by portable biosensing platform, machine vision algorithm analysis in the cloud, and test results obtained via smartphone

To achieve the point-of-care tumor marker detection, an intelligent biosensing platform was designed and fabricated as shown in Fig. 1. Briefly, several prepared reagents are mixed and incubated for luminescent QDs conjugation, the captured images of microfluidic biochips are uploaded to the cloud, and test results are acquired in the smartphone via some relevant machine vision algorithms process and analysis. Among them, the excellent luminescent performance of QDs, functionalized microfluidic biochips, and cloud-based machine vision algorithms are of great significance for this elaborate biosensing platform. Normally, the noninvasive saliva samples containing targeted biomarkers (CEA) are collected from patients, and evenly mixed with the QDs-Ab_1_ and PS-Ab_2_ for conjugation onto the surface of PS microspheres within around fifteen minutes. Next, the conjugated PS microspheres are injected into a designed and fabricated PDMS-based microfluidic biochip with a separation function. Due to this designed function, unconjugated QDs-Ab_1_ will flow through the filtration zone of the biochip and PS microspheres will stay and remain in the filtration zone of the microfluidic biochip. After the UV light irradiation, unconjugated PS microspheres exhibit non-luminescent properties; however, the QDs conjugated PS microspheres present bright green emission as illustrated in the dashed box of Fig. 1. Subsequently, the optical images of microfluidic biochips are captured and uploaded to the cloud through WIFI signal transmission. Then, the related images are recognized and analyzed via some machine vision algorithm for investigation and evaluation of tumor markers. At last, the corresponding test results are acquired in the portable smartphone. Combined with these above capabilities and preponderances, the well-designed intelligent biosensing platform is expected for potential application in point-of-care tumor marker diagnostics fields.

### Morphology and Photoluminescence Property Investigations

**Fig. 2:**
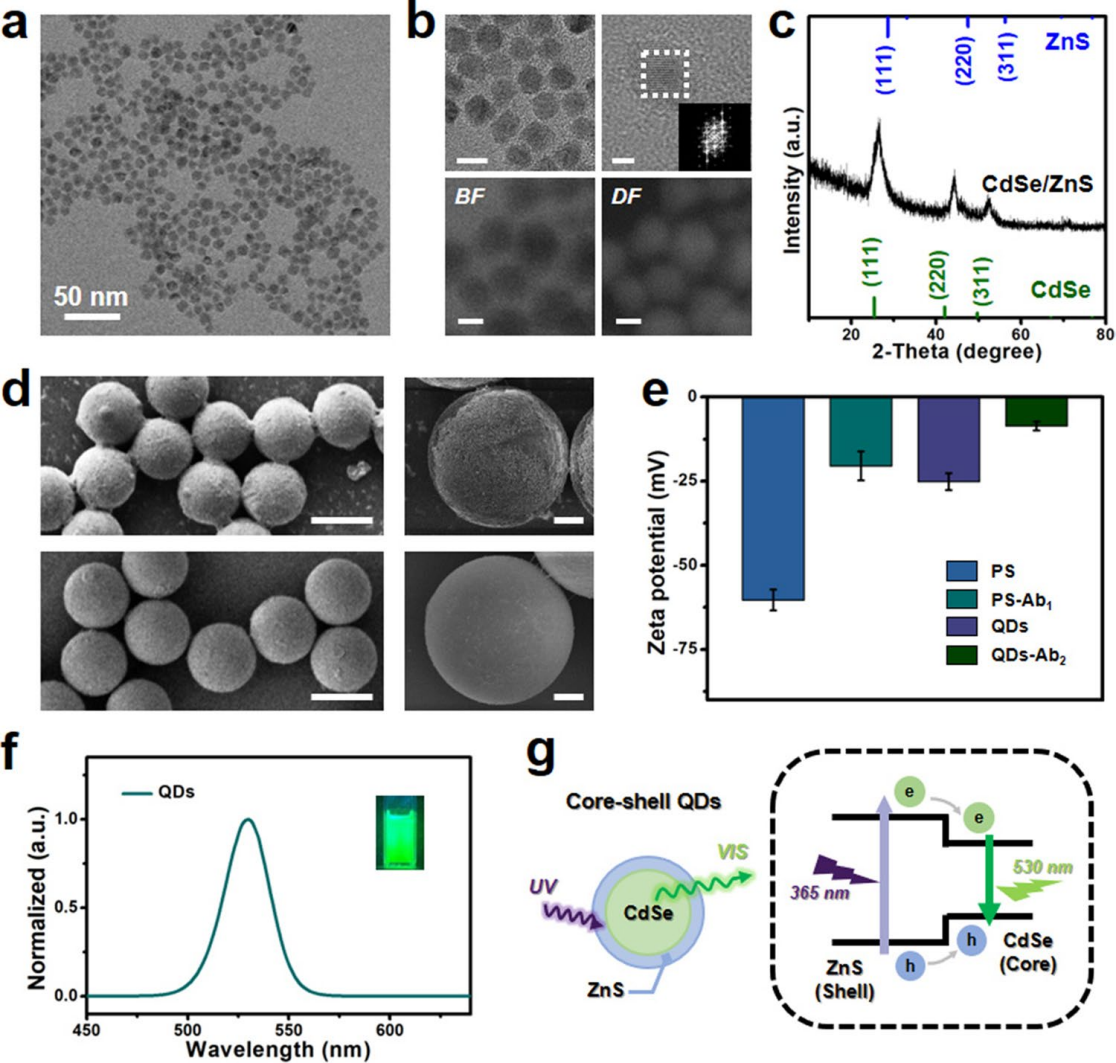
Investigations of morphology, crystalline structure, surface modification, and QDs luminescent property. **a** TEM image of core–shell CdSe/ZnS QDs. **b** HR-TEM images (top) of QDs (scale bar: 10 nm) and relevant inset showing the FFT diffraction pattern (scale bar: 3 nm). STEM images (bottom) under the bright or dark field (scale bar: 5 nm). **c** XRD spectra of core–shell QDs. **d** SEM images of PS microspheres with (up) and without (down) CEA protein conjugation. The left side scale bar is 50 μm, and the right side is 10 μm. **e** Zeta potential histogram of PS microspheres and QDs with and without antibody modification. **f** PL emission spectrum of QDs under UV (365 nm) excitation. The inset is related optical image of QDs. **g** Schematic illustration of the luminescent processes for core–shell QDs under UV irradiation

As shown in Fig. 2, relevant characterizations are studied involving morphology, crystalline structure, surface modification, and QDs luminescent property. The transmission electron microscope (TEM) image of CdSe/ZnS QDs is shown in Fig. 2a, which illustrates that the QDs possess uniform morphology. As shown in Fig. S1, the average particle size of QDs is around 11 nm from the particle size distribution diagram. The top insets of Fig. 2b illustrate the high-resolution transmission electron microscope (HR-TEM) images of CdSe/ZnS QDs. It can be observed that the excellent crystallinity of QDs and the lattice fringes are also clear and distinct, which exhibits the visible fast Fourier transform (FFT) patterns. The related scanning transmission electron microscopy (STEM) images with bright field (BF) and dark field (DF) are investigated as exhibited in the bottom insets of Fig. 2b, which preliminarily indicates the core–shell structure of CdSe/ZnS QDs. Besides, as shown in Figs. S2 and S3, the spectra and mapping of energy dispersive X-ray (EDX) analysis further demonstrate the core–shell form of QDs. The crystalline structure investigation of X-ray diffraction spectrum is shown in Fig. 2c. These obvious diffraction peaks of XRD indicate the core–shell construction of QDs, which is also similar to some previous research works [47, 48].

Moreover, the relevant morphology of PS microspheres is studied in Figs. S4 and  2d. The image of PS microspheres as shown in Fig. S4 illustrates that the microspheres are evenly distributed with a uniform size of around 50 μm. The scanning electron microscope (SEM) images as shown in Fig. 2d further investigate the conjugation situation of QDs onto the surface of PS microspheres with or without CEA tumor markers. It can be observed from the up-insets of Fig. 2d that the QDs are connected to the surface of PS microspheres after related CEA tumor markers conjugation. On the other hand, when there is a bare of CEA tumor markers conjugation, the surface of PS microspheres is relatively clean exhibited in the down-insets of Fig. 2d. As can be observed from Fig. 2e, the investigation of zeta potential additionally demonstrates the modification performance of antibodies onto the surface of QDs and PS microspheres. It can be observed that the values of zeta potential for QDs decreased from −25.2 to −8.6 mV after the connection of antibody. The related zeta potential of PS microspheres also decreases from −60.3 to −20.5 mV because of the antibody modification. The above values variation of zeta potential further indicates that the targeted antibodies are conjugated commendably onto the surface of QDs and PS microspheres. In addition, the relevant photoluminescence performances of QDs are studied in Fig. 2f. Under UV (365 nm) light irradiation, the emission spectrum of CdSe/ZnS QDs is measured at the wavelength range from 450 to 640 nm. It can be observed that the emission peak is located at around 530 nm demonstrating a bright green color emission as exhibited in the inset of Fig. 2f, which implies a potential application for posterior CEA tumor marker detection. The schematic illustration of a probable optical process for CdSe/ZnS QDs is presented in Fig. 2g. When the UV light is exposed to the core–shell CdSe/ZnS QDs, it will emit photons with visible wavelength as shown in the left inset of Fig. 2g. The fundamental optical process starts with the absorption of UV (365 nm) incident photons, in which high energy photons might generate the related electron–hole pairs primarily in the shells of QDs [49]. Subsequently, the created electrons and holes relax fleetly to the band-edge states confining within the core of QDs. Ultimately, the electron–hole pairs recombine radiatively and give rise to the green (530 nm) emission photons [49, 50].

### Microfluidic Biochip with Concentrating and Filtration Performance

**Fig. 3:**
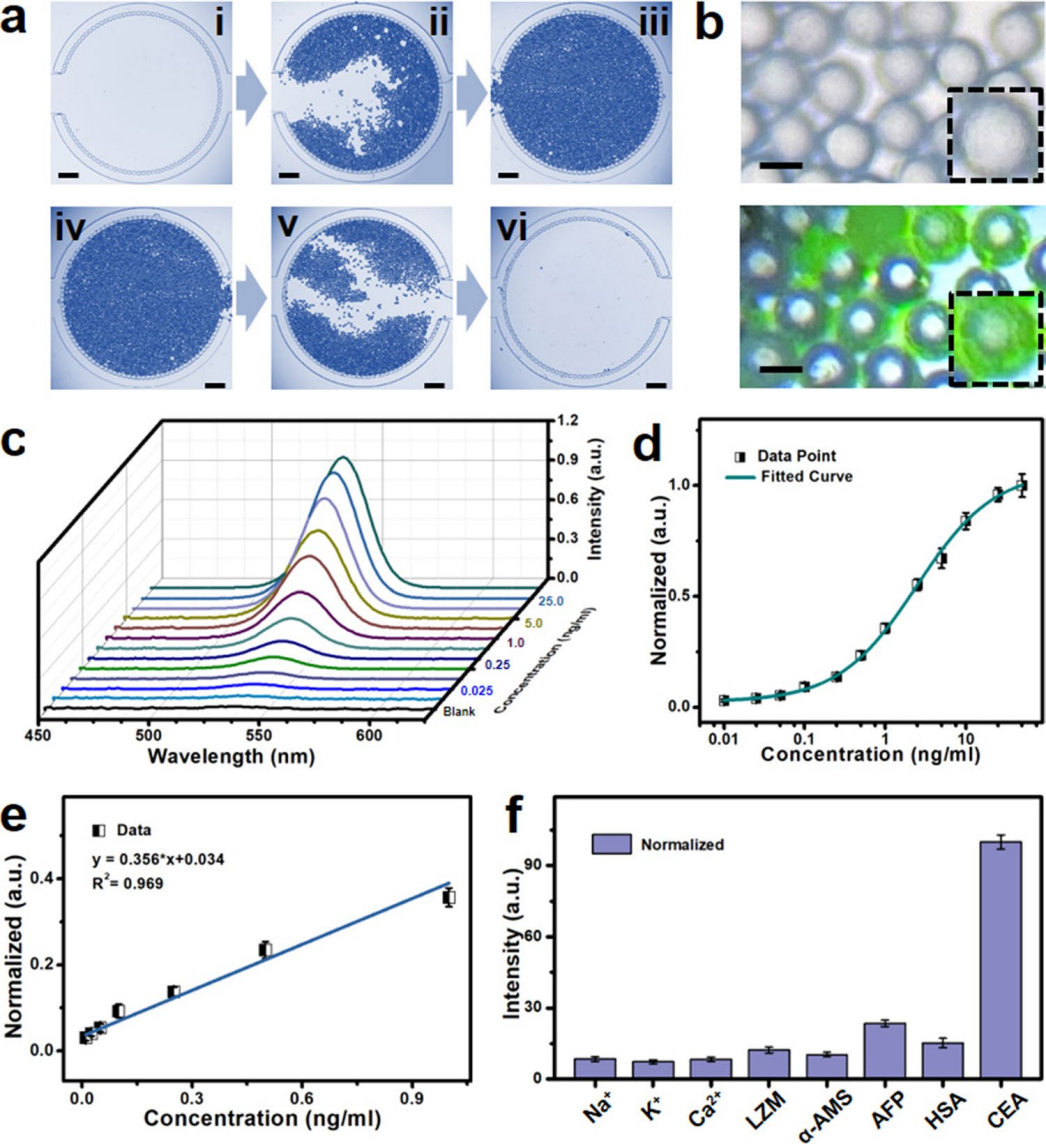
Filtrated and washable capability of microfluidic biochip and related biosensing properties. **a** The filtration (top) and cleanable (bottom) performance images of microfluidic biochip (scale bar: 500 μm). **b** Optical photographs of PS microspheres without (up) and with (down) CEA conjugation (scale bar: 50 μm). **c** The emission spectra of filtrated PS microspheres connected with QDs with different concentrations of CEA conjugation. **d** Normalized intensity of microfluidic biochip with various CEA connections and related fitting curves. **e** The linear relationship for conjugated and separated PS microspheres in microfluidic biochip for CEA protein detection. **f** Specificity test of CEA detection against related interfering objects

The designed and fabricated microfluidic biochips were employed for separating unconjugated QDs and concentrating the luminescent conjugated PS microspheres, which played a vital role in the well-designed biosensing platform for CEA tumor markers diagnostics. As shown in Figs. S5 and S6, the related design layout and optical images of microfluidic biochip indicate that the incubated mixtures will be injected from the inlet and flow to the functional zone subsequently. After that, the unconjugated QDs with the particle size of around 11 nm will be filtrated to the outlet of fabricated microfluidic biochip. On the contrary, the PS microspheres with grain diameter of about 50 μm are retained and concentrated in the separation zone of biochip with filtrated pillar gap of 30 μm. Moreover, the designed microfluidic biochip possesses filtration and cleaning performances as shown in Figs. 3a and S7, S8. Specifically, the related optical images of separation process are illustrated in Fig. S7 and the top insets of Fig. 3a. It can be seen that the PS microspheres are filtrated and concentrated in the filtration zone of microfluidic biochip gradually. From relevant cleaning process images of biochip as shown in the bottom insets of Figs. 3a and S8, the PS microspheres will be washed via injecting of flushing liquid. The corresponding cleanable performance of designed microfluidic biochip brings about the advantage of cost reduction, which improves the application potential for tumor markers detection area. Figures 3b and S9 exhibit the optical images of PS microspheres in the filtration zone of microfluidic biochip with or without CEA tumor markers conjugation. A green luminescence from concentrated PS microspheres can be observed after the CEA conjugation, which further implies the QDs conjugation onto the surface of PS microspheres.

### Diagnostic Properties of Biosensing Platform

In addition, Fig. 3c presents the normalized PL emission spectra with various concentrations of CEA tumor markers. With the increase in CEA concentration, the PL emissions exhibit a rising tendency, which can be attributed to the increased amount of the QDs conjugated onto the surface of PS microspheres. And it can be also observed that the PL intensity increases tardily at the high CEA concentration region. The variation of normalized peak intensity with different concentrations of CEA tumor markers is illustrated in Fig. 3d in the range from 0.01 to 50 ng mL^−1^. The related data points of normalized intensities exhibit a growth trend with the addition of CEA concentration. Among them, the intensity values are normalized under the maximum intensity set as 1.0 standardly. It can be seen that at a low CEA concentration region, the growth trend of normalized intensities tends to be tardiness, which probably originates from the unspectacular QDs conjugations onto the surface of PS microspheres. At the high CEA concentration range, we can also observe a similar trend as the further increase in CEA tumor markers, which can primarily result from the PL intensity saturation of QDs conjugation. As shown in Fig. 3e, the normalized intensity increases linearly with the addition of CEA tumor marker concentration in the range from 0.01 to 1.0 ng mL^−1^. From some previous research, the detection limitation was assessed via concentration related to blank sample plus three times of standard deviation [51–54]. After corresponding calculation, the detection limitation was determined as 0.021 ng mL^−1^ for the fabricated biodetection platform. Compared with corresponding research on CEA tumor marker biodetection, this excellent detection sensitivity implied an advantageous application potential for CEA tumor marker diagnostics [55, 56]. Besides, the relevant investigations of specificity are also significant to evaluate the selection capability of biosensing platform for specific objectives [57–59]. As illustrated in Fig. 3f, some important targets involving Na^+^, K^+^, Ca^2+^, lysozyme (LZM), α-amylase (α-AMS), alpha-fetoprotein (AFP), human serum albumin (HSA), and CEA were tested for specificity study. It can be observed that the normalized intensity of CEA test exhibits a higher value compared with other targets, implying a well diagnostic specificity for this designed CEA tumor marker biosensing platform. The corresponding investigations of tumor marker diagnostic properties demonstrate that this elaborately designed biodetection system possesses enormous potential for future tumor marker diagnostic application.

### Point-of-Care Biosensing Platform with Machine Vision Algorithm

**Fig. 4:**
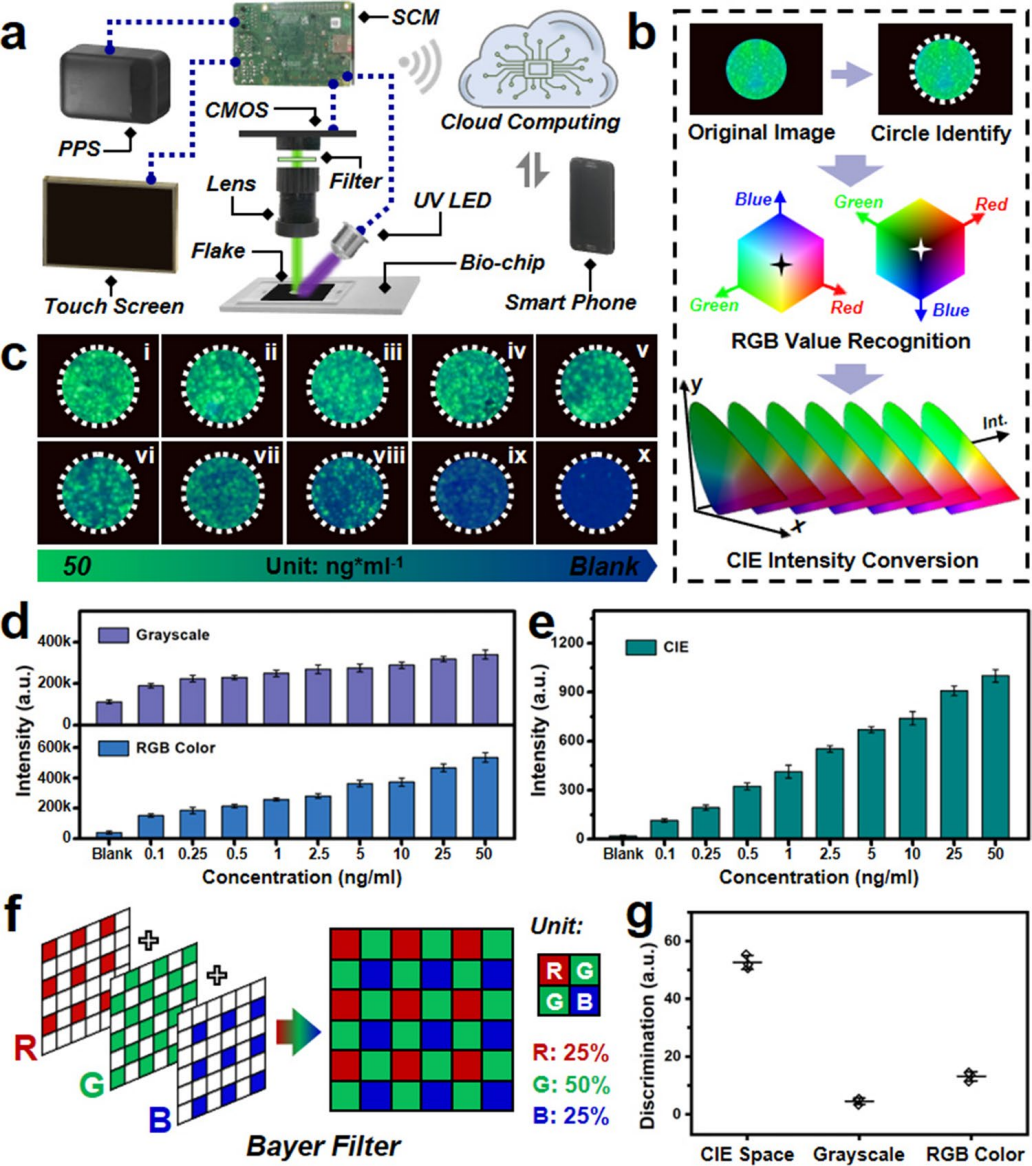
Machine vision algorithm-based point-of-care intelligent biodetection platform. **a** Schematic illustration of point-of-care intelligent biosensing platform with various components. **b** Image recognition workflow of the adopted machine vision algorithm. **c** The optical pictures of microfluidic biochips in separated zones with different CEA concentrations. **d** The calculated grayscale (top) and RGB (bottom) intensity for CEA detection via the intelligent biosensing platform. **e** CIE intensity for this intelligent biosensing system with different CEA concentrations. **f** Schematic diagram of Bayer filter utilized for CMOS electronic components. **g** The discrimination values of image recognition and analysis in various color spaces involving CIE space, grayscale, and RGB color

In order to realize the point-of-care tumor marker diagnostics, an intelligent biosensing platform involving QDs luminescence, microfluidic biochip, and machine vision algorithm was designed and manufactured as shown in Fig. 4. The related schematic illustration of designed intelligent biosensing platform is exhibited in Fig. 4a, which consists of touch screen, portable power supply (PPS), microfluidic biochip, UV (365 nm) LED, black flake, optical lens, light filter, CMOS image sensor, single chip microcomputer (SCM), cloud computing, and smartphone. Among these components of the well-designed biosensing platform, the touch screen was utilized for detection command operation. The PPS with a high battery capacity was selected as the power source for relevant electronic components for a longer running time. The PDMS-based microfluidic biochip was designed and fabricated for separation and concentrating functions. The small accessory of black flake with a circle hole was employed to block some unexpected stray light. The 365 nm UV LED was utilized for incident light providing, which can give rise to a green emission of concentrated and conjugated PS microspheres. Meanwhile, the measurement of absorbance spectra for PDMS-based microfluidic biochip, as exhibited in Fig. S10, indicates that there is almost no absorption for this fabricated biochip in the wavelength of nearby 365 nm, which implies the appropriate usage of PDMS base material for designed microfluidic biochip. In addition, the optical lens and filter were utilized to focus and filter the emission light. The CMOS component was employed for relevant optical image capturing, which possessed several preponderances involving power saving, fast image processing speed, and affordable cost compared with some conventional CCD image sensors. The significant advantage of CMOS image sensor is its power-saving capability over CCD image sensor, which brings about meaningful influence for the application of point-of-care biosensing platform [60]. Besides, the corresponding program management involving data coding, signal processing, and information transmission was carried out in the integrated SCM. The cloud computing was utilized for executing related machine vision algorithms and the corresponding test results of tumor marker diagnostics were obtained via the smartphone mobile terminal. The workflow chart of this designed intelligent biosensing platform is illustrated in Fig. S11 for CEA tumor marker detection. Among them, the CMOS image sensor is one of the significant components, which converts optical signals to electronic signals. Then, these obtained images will be uploaded to the cloud by an integrated WIFI module for further photo-processing. The reason for this design is that it can reduce the related cost of portable devices for image processing and analysis because they are relatively expensive for the SCM and corresponding electronic accessories with eminent image processing ability and interaction capability with mobile terminals. On the other hand, it will also take up a large space for biosensing platform, which restricts the point-of-care applications for tumor markers diagnostics. In that case, the crucial procedure of image processing was implemented in the cloud, which will bring about excellent benefits like cost reduction and efficiency improvement.

For the purpose of accomplishing point-of-care tumor marker diagnostics, the Python-based machine vision algorithm was developed and employed for acquired image recognition and processing. As shown in Fig. 4b, the original image was processed for luminescent circle identification via the algorithm. Subsequently, this Python-based machine vision algorithm will automatically recognize and extract the related luminescent points RGB values in the identified circle image. Ultimately, the extracted RGB values in RGB color cubic space will convert into CIE values (x, y, Y) for further image analysis, which manifests that each recognized luminescent circle image corresponds to a data point in the CIE xyY color space. According to previous research works, the luminance and intensity of emission light are relatively equivalent in the CIE xyY color space, and the chromaticity values (x, y) are determined by the emission light wavelength [61, 62]. Due to the independent characteristic for luminance and wavelength of emission light, the CIE intensity (Y) will not be influenced by the chromaticity values (x, y) of CIE xyY color space [61]. Regarding this designed QDs luminescence sandwich immunoassay structure for CEA tumor marker detection, the wavelength and intensity of emission spectra are mutually independent, as illustrated in Fig. 3c, d, the intensity of emission spectra changes with the variation of CEA tumor marker concentrations. In that case, the CIE intensity (Y) value was employed for evaluating the microfluidic biochip image signal intensity performance. The positive (with CEA proteins) samples and negative (without CEA proteins) samples were investigated in this designed intelligent biosensing platform as shown in Figs. S12–S14, and related captured optical images were processed and analyzed via the Python-based machine vision algorithm. As exhibited in Fig. S12, it can be observed that negative samples and positive samples were automatically recognized via this machine vision algorithm. The positive samples show bright green emission; nevertheless, the negative samples exhibit unobvious emission light. Besides, the calculated average chromaticity values (x, y) of CIE xyY color space were illustrated, which demonstrates that the positive and negative samples possess disparate chromaticity values. These relevant and thrilling investigations imply that the Python-based machine vision algorithm can be utilized for the intelligent biosensing platform of CEA tumor marker detection.

Furthermore, a series of microfluidic biochip luminescent images were obtained in the intelligent biosensing platform and processed by the machine vision algorithm for CEA tumor marker diagnostics. As shown in Fig. 4c, it can be observed several optical images with different concentrations of CEA tumor marker conjugation. With the decrease in concentrations, the luminescence intensity illustrates a declining trend. By means of the grayscale and RGB color space investigations for these various obtained images, as shown in Fig. 4d, the related intensities indicate a growth trend as the addition of CEA tumor marker concentrations. Among them, the corresponding intensities of grayscale investigation increase relatively tardily with the increase in CEA tumor marker concentrations. The relevant intensities variation trend of G values for RGB color space exhibits a better rising tendency but the related linear relationship manifests relatively poor as the growth of CEA tumor marker concentrations. On the other hand, as shown in Fig. 4e, the calculated CIE intensities from the machine vision algorithm exhibit better increase trend and quantitative relationship at the range from blank to 50 ng mL^−1^of CEA tumor marker concentrations. The concentration cut-off value is determined as a relatively low intensity of 0.1 ng mL^−1^, which implies a potential application for CEA tumor marker detection. Besides, as mentioned above, the CMOS component is significant for the optical images obtained. Among this electronic component, the Bayer filter is commonly used in the commercial CMOS image sensor. The related schematic illustration is presented in Fig. 4f, and it can be observed that the Bayer filter involves three primary colors of red, green, and blue. The proportion of green color in a unit is 50%, twice than the other colors, which can be attributed the similar color sensitivity with human eye [63, 64]. This is also one of the other reasons for the selection of green emission QDs as a luminescent agent in the intelligent biosensing platform for CEA tumor marker diagnosis. In addition, the discrimination values of three different image analysis are investigated in Fig. 4g. The corresponding discrimination values were calculated from the ratio of luminescent intensities with blank samples and highest concentration samples. It can be observed that the relevant discrimination value of this algorithm system is much higher than the grayscale and RGB color space methods, which implies the suitable adoption for the Python-based machine vision algorithm. Therefore, combined with all these corresponding advantages and performances, this well-designed intelligent biosensing platform is of great importance for future point-of-care tumor marker diagnostics.

### Diagnostic Properties Comparison with LFA Strips

**Fig. 5:**
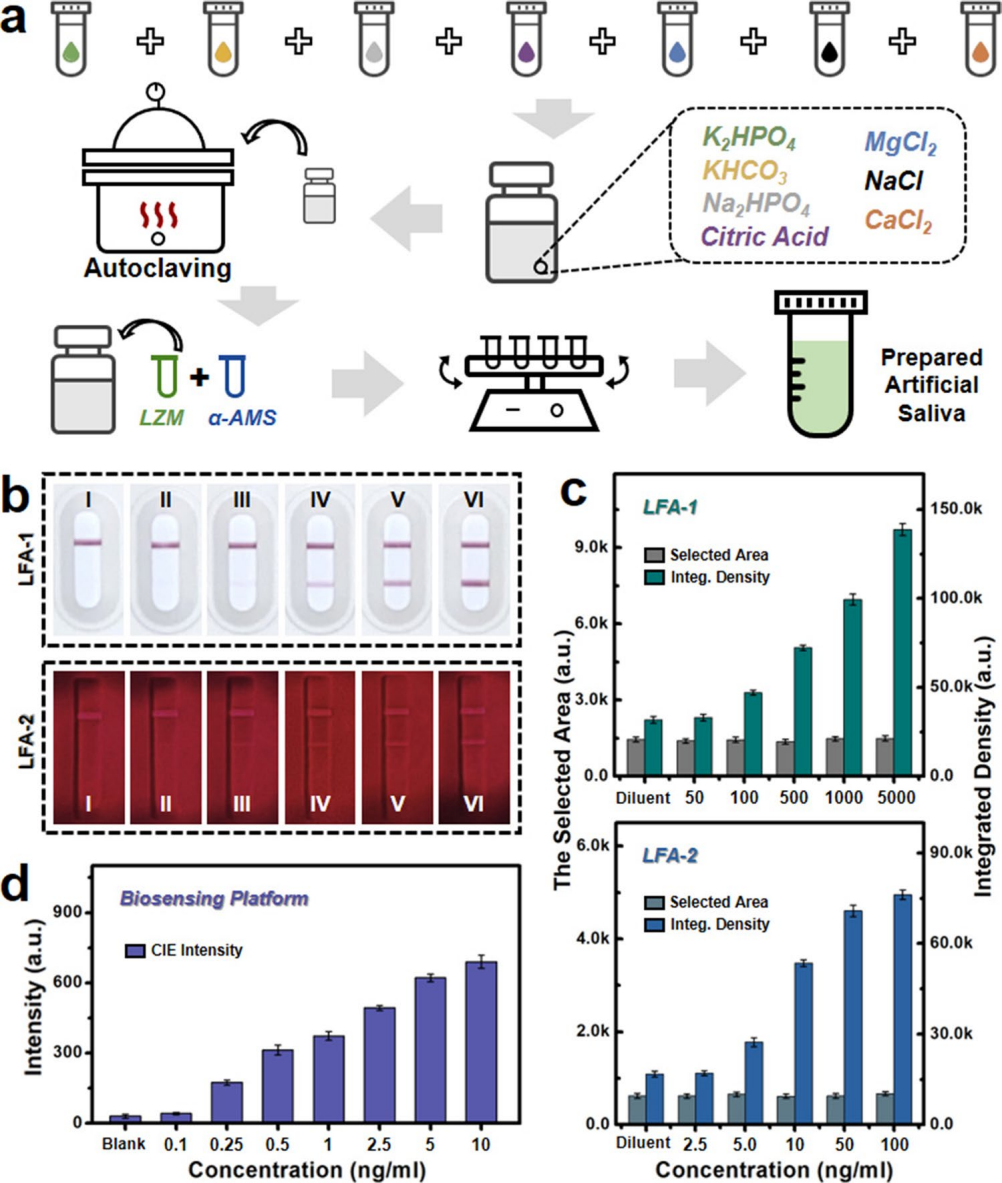
Detection performance comparison for simulated samples of CEA tumor marker. **a** Schematic diagram for artificial saliva preparation. **b** The optical images of two commercial CEA tumor marker test strips including LFA-1 (top) and LFA-2 (bottom). **c** Histogram plot of related commercial test strips with LFA-1 (up) and LFA-2 (down) by gray values calculated from the selected area. **d** The column chart of CIE intensity for designed biosensing platform with diverse CEA tumor marker concentrations

Moreover, the potentially practical diagnostic performance was investigated for this designed intelligent point-of-care biosensing platform, and a series of test samples involving purified human source CEA proteins and synthetic saliva solvent were employed for detection capabilities evaluation as shown in Fig. 5. For the purpose of studying practical applications, the prepared artificial samples were utilized for simulating the clinical samples with concentration levels, matrix solvent composition, and other relevant parameters. The schematic diagram of procedures for artificial saliva solvent preparation is illustrated in Fig. 5a according to previous studies [65, 66]. In detail, the 5 mL volume of several aqueous solutions involving K_2_HPO_4_ (25 mM), KHCO_3_ (1.57 M), Na_2_HPO_4_ (24 mM), MgCl_2_ (2 mM), and NaCl (0.1 M) were mixed with 5 mL of CaCl_2_ (0.15 M) and 3 mL of citric acid (25 mM) adequately. Then, the NaOH and HCl were utilized to adjust the pH to around 7.0. Next, the volume of the mixture aqueous solution was expanded to 50 mL quantitatively. Subsequently, the enlarged mixture solution was sterilized via the autoclaving method. After the sterilized solution reached room temperature, 0.5 mL of aqueous solutions including α-AMS (1 g L^−1^) and LZM (0.1 g L^−1^) were added into the sterilized solution and continuously mixed for around half an hour. Ultimately, various concentrations of human source CEA tumor marker proteins were blended with the prepared artificial saliva for further detection performance investigations.

As shown in Fig. 5b, the related optical images for two commercial LFA strips are illustrated with various CEA protein additions. Among them, the top images exhibit the colorimetric LFA strips (LFA-1) with colloidal gold labeling, and the fluorescent LFA strips (LFA-2) are displayed in the bottom of Fig. 5b. It can be observed that the test line of LFA strips present fuzzy and unclear performance with the decrease in CAE protein concentration from insets of (VI) to (I). In addition, the corresponding principles of commercial LFA are illustrated in Fig. S15. Normally, the relevant functional areas are involved in the commercial LFA including the sample area, conjugated area, test/control lines, and absorbent area. The test sample consisting of targeted antigens is dropped in the sample area and then laterally flowed to the conjugated area for the corresponding conjugation with specific antibody-modified tracing agents. Next, the conjugated tracing agents will sequentially flow to the areas of antibodies modified test/control lines for sandwich immunoassay. Finally, the related residuums involving unconjugated tracing agents and buffer solvent laterally flow to the absorbent area. Ulteriorly, to further quantitatively analyze the detection properties of commercial LFA strips, the related optical pictures were converted into the corresponding grayscale images exhibited in Figs. S16 and S17. As shown in Fig. 5c, the test line selected areas of relevant LFA strips were managed basically at the parallel area values for better comparison of diagnostic capabilities. Regarding LFA-1 test strips, the values of integrated density tend to descend as the decrease in CEA protein concentrations. The relevant LFA strips indicate a relatively poor diagnostic property, and the detection cut-off value is estimated at about 100 ng mL^−1^, which is similar to the correlative optical images shown in Fig. S16 and the top group insets of Fig. 5b. In terms of LFA-2 strips, the corresponding values of integrated density indicate decrease trend as the reducing addition of CEA concentrations as well, which demonstrates a diagnostic cut-off value of around 5.0 ng mL^−1^. In addition, as shown in Figs. 5d and S18, the designed intelligent biosensing platform was also employed to investigate the relevant CEA tumor marker saliva samples diagnostic performance. Similarly, with the increase in CEA protein concentrations, the corresponding values of CIE intensity show a growth trend. After related machine vision algorithm processing, the cut-off value is assessed at about 0.25 ng mL^−1^, which is better than the tested commercial LFA strips for tumor markers point-of-care diagnostics. Besides, the corresponding linear relationship for this biosensing platform is investigated as shown in Fig. S18, which exhibits a great linear dependence property for CEA tumor marker concentrations. This well-designed intelligent biosensing platform reveals tremendous advantages, which is expected for thrillingly potential application for point-of-care tumor markers diagnostics in the future.

## Conclusion

In summary, an intelligent point-of-care biosensing platform based on microfluidic biochip and machine vision algorithm was developed and manufactured for CEA tumor marker detection. The uniform core–shell QDs of CdSe/ZnS with extraordinary luminescent properties were employed for the optical labeling indicator of the designed sandwich structure immunoassay. The elaborate microfluidic biochip with excellent filtrated and cleanable performance was utilized as a functional chamber accessory of the designed CEA tumor marker biosensing platform, which illustrated a prominent diagnostic sensitivity of around 0.021 ng mL^−1^. The Python-based machine vision algorithm was developed and applied as the corresponding image recognition and analysis for expected point-of-care CEA tumor marker detection. Moreover, for the investigation of saliva samples practical application evaluation, the designed intelligent biosensing platform exhibited outstanding detection cut-off value compared with some commonly used commercial CEA tumor marker LFA test strips. As a result, considering these thrilling and remarkable system design superiority and diagnostic abilities, this well-designed intelligent biosensing platform implies tremendous application potentiality for the future point-of-care tumor marker detection field.

## Supplementary Information

Below is the link to the electronic supplementary material.

Supplementary file1 (DOCX 1678 KB)
