# Supplementary material for: Intelligent Point-of-Care Biosensing Platform Based on Luminescent Nanoparticles and Microfluidic Biochip with Machine Vision Algorithm Analysis
Source: Nanomicro Lett. 2025 Apr 14;17:215. doi: 10.1007/s40820-025-01745-w (PMC11996743; doi:10.1007/s40820-025-01745-w)
Supplement: Supplementary file 1 — Supplementary file1 (DOCX 1678 KB) [file 40820_2025_1745_MOESM1_ESM.docx]

Supporting Information for

**Intelligent Point-of-Care Biosensing Platform Based on Luminescent Nanoparticles and Microfluidic Biochip with Machine Vision Algorithm Analysis**

Yuan Liu^1^, Xinyue Lao^1^, Man-Chung Wong^1^, Menglin Song^1^, Yifei Zhao^1^, Yingjin Ma^1^, Qianqian Bai^1^, Jianhua Hao^1,2^*

^1^ Department of Applied Physics, The Hong Kong Polytechnic University, Kowloon, 999077, Hong Kong, P. R. China

^2^ Research Centre for Nanoscience and Nanotechnology, The Hong Kong Polytechnic University, Kowloon, 999077, Hong Kong, P. R. China

*Corresponding author. E-mail: [jh.hao@polyu.edu.hk](mailto:jh.hao@polyu.edu.hk) (Jianhua Hao)

**S1 Experimental Section**

***Chemicals and Materials:*** 1-Ethyl-3-(3-dimethylaminopropyl)-carbodiimide hydrochloride (EDC) and N-hydroxysulfosuccinimide sodium salt (sulfo-NHS) were obtained from TCI. Carboxyl-modified CdSe/ZnS QDs were purchased from Xiamen Bohr Technology Co., Ltd. Carboxyl-modified monodisperse polystyrene (PS) microbeads were purchased from Wuxi Rigor Technology CO., LTD. Carcinoembryonic antigen (CEA) and CEA antibodies were purchased from GenScript Biotech Corporation. All the above chemicals were used without further purification.

***Characterizations:*** A field emission transmission electron microscopic (TEM) (JEOL JEM-2100F) was used to acquire TEM images. Scanning electron microscope (SEM) images were obtained using a Tescan MIRA field emission SEM. The X-ray diffraction (XRD) was characterized by an X-ray Diffractometer (Rigaku SmartLab). Zeta potential was recorded by a Malvern Zeta Potential Analyzer. Optical photos of PS microspheres were captured by Leica DM1750 optical microscopy. The EDINBURGH fluorescence spectrometer was used to measure the relevant emission spectra. A PERKIN ELMER UV-Vis-NIR spectrometer was used to acquire the absorbance spectra.

***Conjugation of QDs with antibody:*** The conjugation of antibodies with CdSe/ZnS QDs or PS microbeads was carried out by traditional EDC/NHS cross-linking methods. EDC (3 mg) and NHS (6 mg) were added to the carboxyl-modified CdSe/ZnS QDs (1 mL, 1 mg/ml) and stirred for 30 min. Then, 0.1 mg of CEA antibody was added to CdSe/ZnS QDs and stirred for another 2.5 h to ensure a complete coupling reaction. The antibody conjugated QDs were collected by high-speed centrifugation and washed with water three times.

***Conjugation of PS microspheres with antibody:*** The 10 μL of 10 mg/mL EDC/NHS was added into 1mg of carboxy group modified PS microbeads and the solution was stirred for 30 min. Then the PS nanobeads were precipitated and redispersed in PBS, with the addition of 0.3 mg of CEA antibody in 800 μL PBS. The mixture was incubated for 2.5 h. The resultant antibody conjugated PS microbeads were washed and resuspended in PBS for further usage.

***Microfluidic biochips fabrication:*** The utilized microfluidic biochips were manufactured by the conventional soft lithography methods. Specifically, the pattern was designed through the AutoCAD software and then manufactured to a film mask. In terms of the specific process of wafer mold, the 4-inch silicon wafer was cleaned via acetone, IPA, DI Water, and N_2_ gas, respectively. The SU-8 photoresist (~4 ml) was spun coating onto the surface of the wafer with no bubble. Next, the photoresist coated wafer was treated for soft bake under the two temperature of 65/95 degrees Celsius and follow by pattern transfer via the common photoetching machine. Subsequently, the post bake was carried out with the same temperature 65/95 degrees Celsius and the baked wafer was executed for developing with SU-8 developer, IPA, DI Water, and N_2_ gas in sequence. After the hard bake for 150 degrees Celsius, the wafer was cooling down to room temperature. In the second step, the PDMS-glass bonded biochip was fabricated via the followed steps: the fabricated wafer mold under the lithography approach was evaporated with a thin organic film for better demolding, and follow by the PDMS pouring and thermocuring with the ratio of 1:10 (curing agent and base). Then, the cured PDMS with designed pattern was implemented with demolding and clipping for the following plasma treatment bonding. The plasma treated PDMS chips and glasses were bonded and heated for 85 degrees Celsius with 5 minutes. Ultimately, the biochips were successfully manufactured after the channels cleaning by N_2_ gas.

**S2 Supplementary Figures**


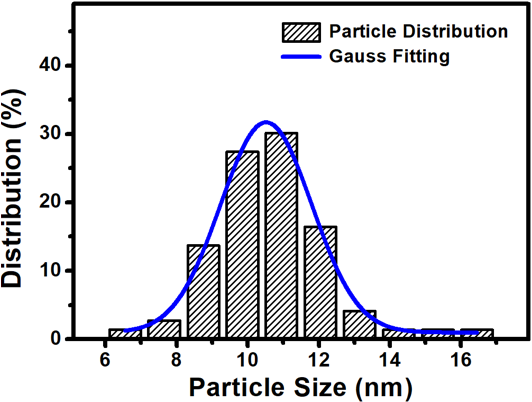


**Fig. S1** The particle sized distribution histogram of CdSe/ZnS QDs and related Gauss fitted curve


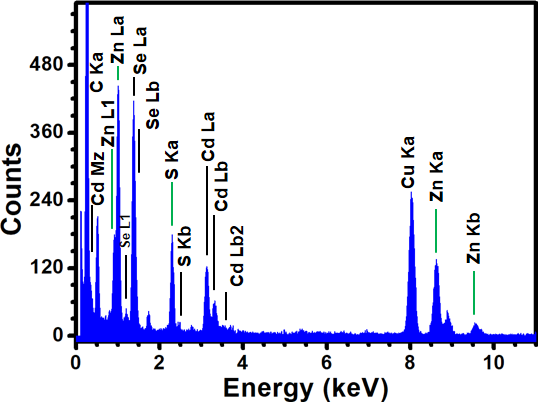


**Fig. S2** EDX spectra of core-shell CdSe/ZnS QDs in TEM samples


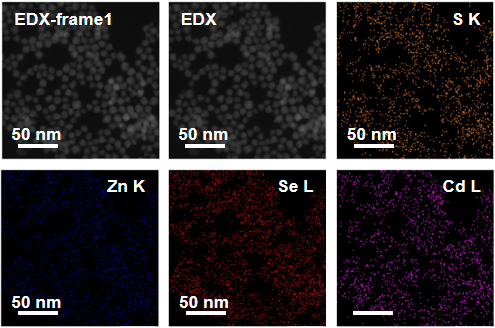


**Fig. S3** The related EDX elements mapping of CdSe/ZnS core shell QDs


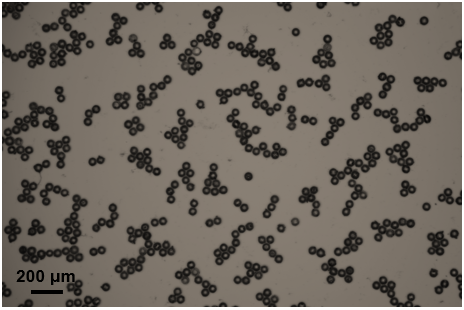


**Fig. S4** The relevant image of PS microspheres under the reflection mode of light microscope


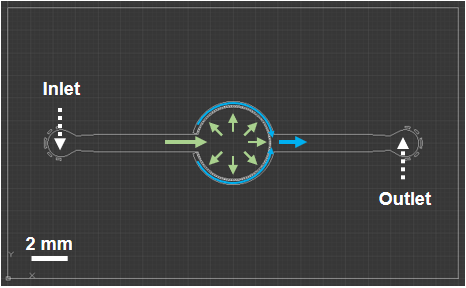


**Fig. S5** The layout of AutoCAD design for prepared microfluidic biochip and related flow direction


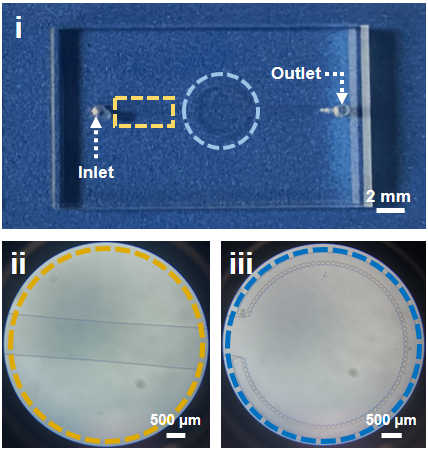


**Fig. S6** The optical photo of fabricated PDMS microfluidic biochip (i), related microscale images of flow channel (ii), and concentrating area (iii)


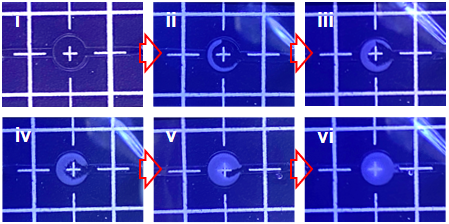


**Fig. S7** The pictures of microfluidic biochip concentrating property from (i) to (vi) insets


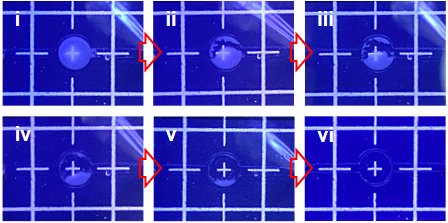


**Fig. S8** The photos of cleaning performance for microfluidic biochip from (i) to (vi) insets


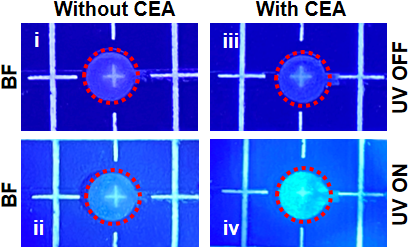


**Fig. S9** The corresponding optical image for microfluidic biochip with or without CEA conjugation under the UV-on or UV-off in bright field


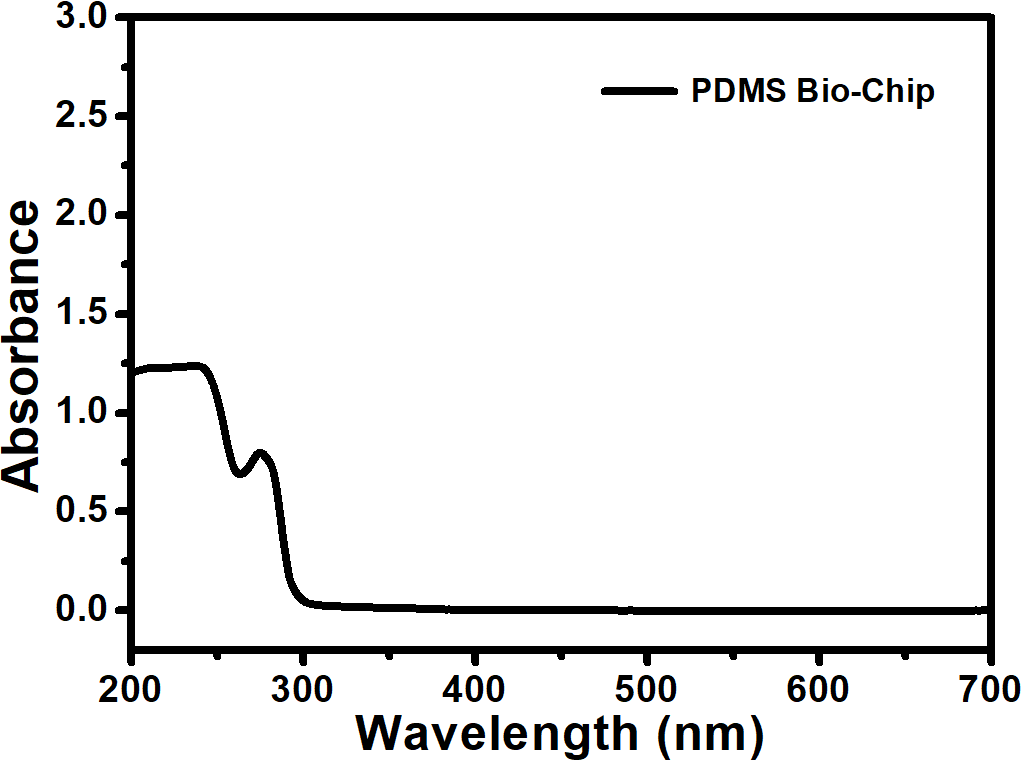


**Fig. S10** The absorbance spectrum of PDMS-based microfluidic biochip


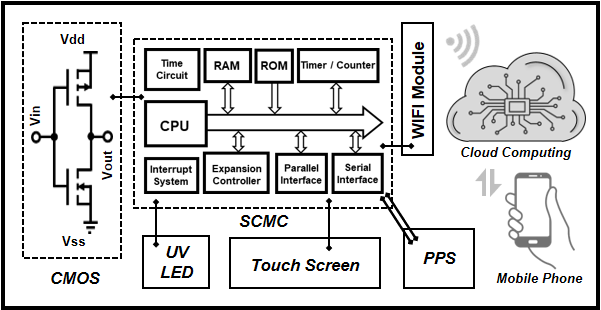


**Fig. S11** The flow chart of the manufactured biosensing platform for CEA detection combined with cloud computing and mobile phone results obtain


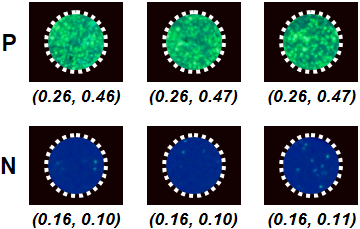


**Fig. S12** The acquired optical images of CEA tumor marker positive (P) or negative (N) samples and their corresponding average CIE (x, y) values


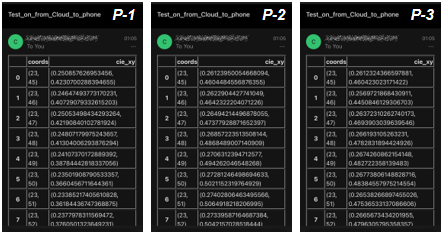


**Fig. S13** The smartphone screen pictures of designed biosensing platform for relevant positive (P) samples CIE (x, y) values performance


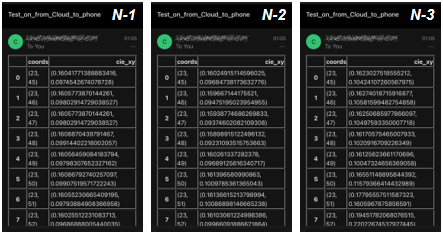


**Fig. S14** The mobile phone screen photos of designed biosensing platform for related negative (N) samples CIE (x, y) values performance


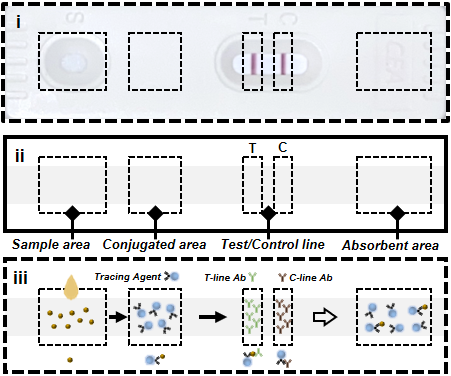


**Fig. S15** The picture of commercial rapid test strip (i), the corresponding functional area illustration (ii), and schematic diagram of lateral flow assay principles (iii)


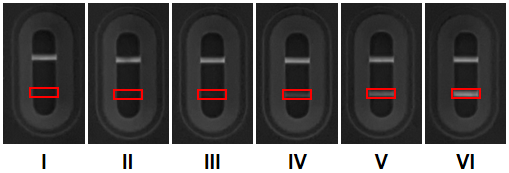


**Fig. S16** The converted grayscale photos of colorimetric (LFA-1) strips for saliva CEA tumor marker detection


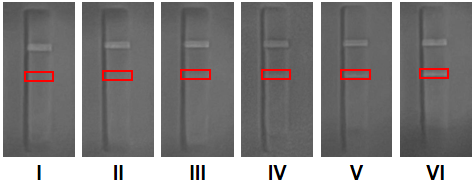


**Fig. S17** The converted grayscale pictures of fluorescent (LFA-2) strips for saliva CEA tumor marker diagnostics


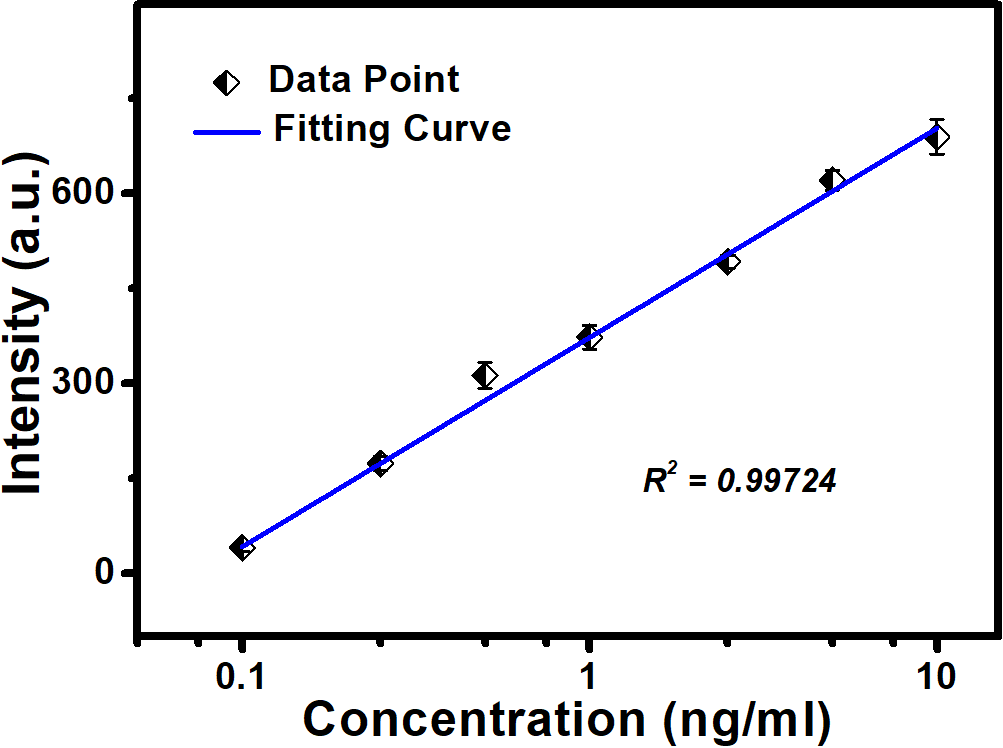


**Fig. S18** The linear relationship with the designed biosensing platform for saliva CEA tumor marker detection
